# Supramolecular Nanomedicines of *In-Situ* Self-Assembling Peptides

**DOI:** 10.3389/fchem.2022.815551

**Published:** 2022-02-04

**Authors:** Ying Zhang, Yingying Yu, Jie Gao

**Affiliations:** State Key Laboratory of Medicinal Chemical Biology and College of Life Sciences, Nankai University, Tianjin, China

**Keywords:** peptides, supramolecular, *in situ*, self-assambly, nanomedicines

## Abstract

Nanomedicines provide distinct clinical advantages over traditional monomolecular therapeutic and diagnostic agents. Supramolecular nanomedicines made from *in-situ* self-assembling peptides have emerged as a promising strategy in designing and fabricating nanomedicines. *In-situ* self-assambly (SA) allows the combination of nanomedicines approach with prodrug approach, which exhibited both advantages of these strategies while addressed the problems of both and thus receiving more and more research attention. In this review, we summarized recently designed supramolecular nanomedicines of *in-situ* SA peptides in the manner of applications and design principles, and the interaction between the materials and biological environments was also discussed.

## 1 Introduction

Nanomedicine utilizes nanotechnology to treat or prevent human diseases. Nanomedicine-based drug delivery systems often show enhanced therapeutic efficacy, lower toxicity and improved bioavailability and selectivity than conventional free drugs (Gao et al., 2012; Oberoi et al., 2013) because of their sizes (10–100 nm), large amount of drug loading and targeted surface functionalization. (Xie et al., 2010; Blanco et al., 2015; Lim et al., 2015; Shi et al., 2017; Sato et al., 2018). Many nanomedicines have already been approved for clinical use or are undergoing clinical trials. However, for now, the successful translation of nanomedicine in the clinic remains challenging. (Venditto and Szoka, 2013). The poor penetration and transportation of nanomedicine through biological barriers *in vivo* are two most significant factors delaying the clinical translation. (Heldin et al., 2004; Flessner et al., 2005; Matsumoto et al., 2016).

Prodrug is another important strategy that have been widely used to address delivery problems with therapeutic agents. (Chakroun et al., 2019; Zhou et al., 2019; Li et al., 2020; Wang et al., 2020). Just like nanomedicine approaches, its goal is to improve the selectivity and to increase the concentration of drugs at desired locations. Prodrug focuses on improving physicochemical properties allow for enhanced permeability and solubility, by which it could finally achieve tissue-specific drug delivery and reducing toxic side effects. However, prodrugs still suffered from many drawbacks related to conventional free drugs such as poor chemical stability and quick clearance *in vivo*.

In recent years, *in-situ* self-assembly (SA) of drug-peptide conjugates, which integrated the approaches of nanomedicine and prodrug, have emerged as a promising strategy in targeted delivery of therapeutic agents and diagnostic molecules. (Wang et al., 2017; Liang et al., 2020; Schiapparelli et al., 2020). The drug-peptide conjugates firstly served as prodrugs and would then fabricate into supramolecular nanomedicines by site-selective stimulus, including factors in microenvironment such as different ionic strengths or pH (Cong et al., 2019; Kubota et al., 2020) and temperatures, (Nasrollahi et al., 2018; Ji et al., 2019), upregulated redox molecules namely glutathione, (Xu et al., 2018), hydrogen peroxide and some other reactive oxygen species, (Miao et al., 2013; Wu et al., 2019), and abnormally expressed enzymes like esterases, proteases and phosphatases, (Li et al., 2015; Shi et al., 2018; Wang H et al., 2019), such *in-situ* SA approach gained success in enhancing the permeability of nanomedicine and increasing the concentration of therapeutic agents at disease sites. (Cai et al., 2017; Zhang X et al., 2018; Cheng et al., 2019a; Feng et al., 2019; Zhou et al., 2019).

Supramolecular nanomedicine of *in-situ* SA peptides is a very promising research area. It has the potential to become a key field in nanomedicine and chemical biology because it attempts to combine the precise molecular control of synthetic material with the complexity of biology. In this review, two main categories of applications are identified and discussed for *in-situ* SA peptides, namely drug delivery and diagnosis. Key concepts such as molecular design, synthetic routes and the interaction between the materials and biological environments will also be discussed.

## 2 *In-situ* SA Peptides for Targeted Drug Delivery

Previous studies have shown that, pre-assembled nanomaterials *in vitro* may face the problems of reduced bioavailability, liver and spleen toxicity and side effects because nano-fractures can be easily captured by macrophages system. (Chien et al., 2013). Therefore, in recent years, researchers have begun to construct materials from *in-situ* SA short peptides that respond to special stimuli in living cells and animals, and achieve biological functions by fine-tuning the structure of short peptides and assembly strategies. (Deng et al., 2021). The pathological environment is usually different from normal tissue, which may be manifested in the following aspects: pH (Boedtkjer and Pedersen, 2020), enzymes (Shahriari et al., 2019), reactive oxygen species (ROS) (Aggarwal et al., 2019) and glutathione (Yang B et al., 2019). Under those pathologic conditions, the targeted construction of self-assembly supramolecular was possible. Cancer (malignant tumor), a malignant disease that threatens human life and health, is characterized by the rapid growth and metastasis of tumor cells. Systemic radiotherapy and chemotherapy are not targeted to cancerous tissues, and often produce toxicity to normal tissues and cause serious side effects. Therefore, it is very important to seek targeted delivery therapy. Targeted therapy has the following advantages: specific target, low side effects and individualized treatment. Considering investigational and approved nanomedicine, the most prominent area of current use is in the treatment of cancers, where drug targeting is a major issue. In the following section, we summarized the application of *in-situ* SA peptides in the treatment of cancer and categorized them by stimulus that activated their SA.

### 2.1 pH

**FIGURE 1:**
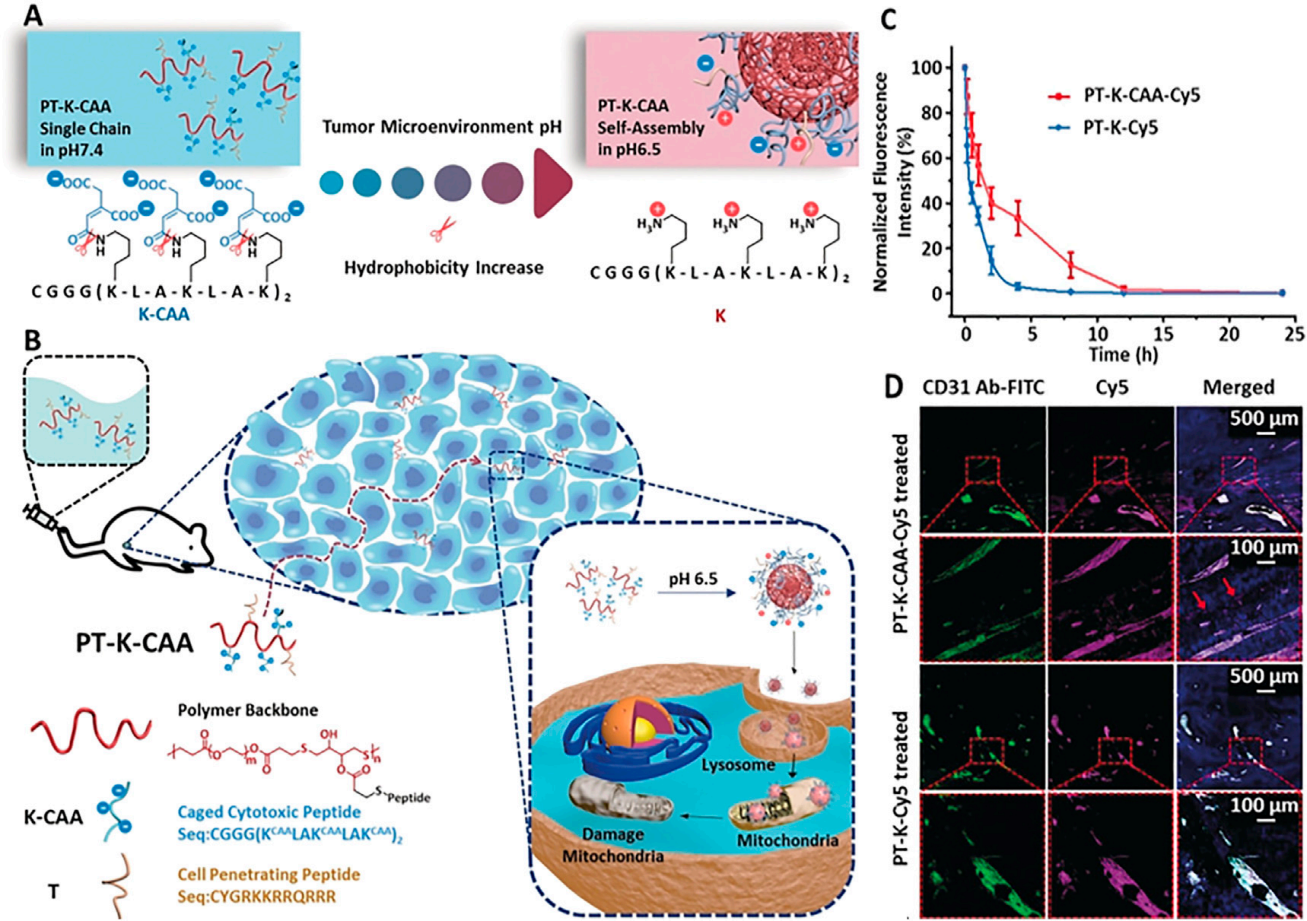
**(A)** Process of PT-K-CAA hydrolysis, which leads to its self-assembly **(B)** Process for the penetration of PT-K-CAA in the tumor microenvironment **(C)** Normalized Cy5 FL of the blood collected at different times after tail-vein injection of Cy5-labeled PPCs **(D)** Frozen sections of B16F10 tumors after treatment with Cy5-labeled PT-K-CAA or PT-K. Nuclei and tumor vessels were stained with 4′,6-diamidino-2-phenylindole (DAPI) and fluorescein isothiocyanate (FITC)-tagged CD31 antibody, respectively. Copyright 2019, Angewandte Chemie-International Edition.

In the process of glycolysis, cancer cell will produce a mass of hydrogen ions, pyruvate and lactic acid, which shape the ‘acidic physique of tumors’, and the pH value of the tumor microenvironment fluctuates between 5.5–6.5. (Rohani et al., 2019). Wang’s group designed a polymer-peptide conjugates (PPCs) named PT-K-CAA. (Cong et al., 2019). PT-K-CAA was obtained by coupling two polypeptides with the main chain β-thioester. two kinds of peptides were obtained, one was a therapeutic peptide which was modified by a pH-sensitive unit *cis*-aconitic anhydride (CAA), and the other was a cell penetrating membrane peptide (Figure 1B). At pH 7.4, PT-K-CAA remained as single chains and it self-assembles into nanoparticles at pH 6.5 due to the hydrolysis of the CAA groups (Figure 1A). They first used multicellular spheroids (MCSs) to verify the high tumoral permeability of PT-K-CAA (Figure 1D). PT-K-CAA (red signals) could be observed, on the contrary the vessels (green signals) were missing (red arrows; Figure 1C), which indicated its excellent penetrability. In addition, PT-K-CAA was negatively charged, which might prolong its circulating time. In BALB/c mice, the half-life of PT-K-CAA was up to about 2 h (Figure 1C). In another work by Wang and co-workers, a hierarchical responsive nanomedicine (HRNM) was designed for programmed delivery of chemotherapeutic drug. (Wang et al., 2018). In blood circulation, RGD peptide was protected by POEG, therefore HRNMs with nanometer size could achieve effective tumor enrichment through passive targeting. Once HRNMs reached the tumor site, the RGD peptide will be exposed due to the acidic tumor microenvironment induced the conversion of the hydrophilicity of the PC7A chain to enhance tumor retention and cell internalization. These two works suggested that it is effective to use the weak acidity inside the tumor to achieve *in-situ* SA.

### 2.2 Enzymes

#### 2.2.1 Alkaline Phosphatase

**FIGURE 2:**
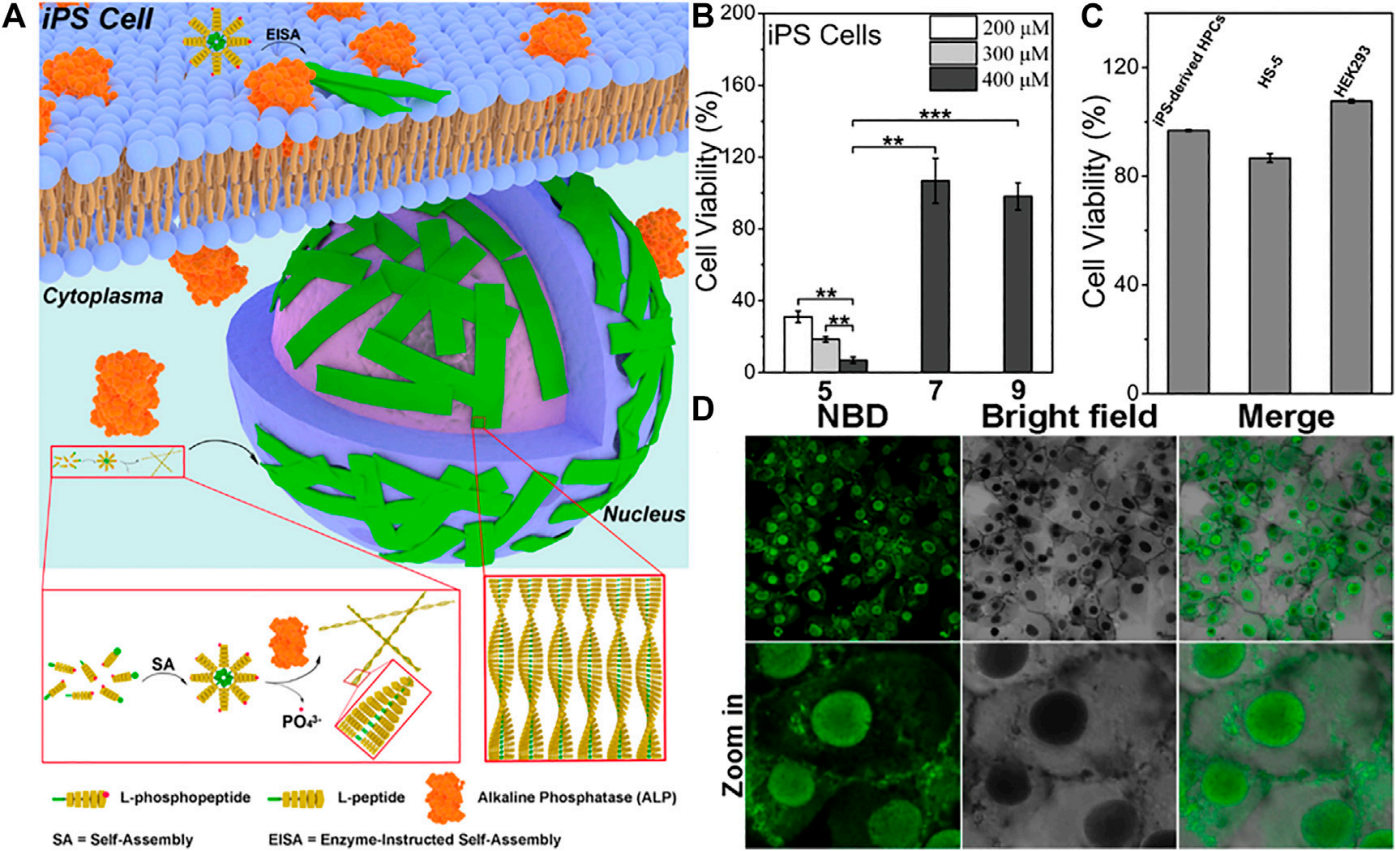
**(A)** Schematic representation of EISA of l-phosphopentapeptide 5 to result in intranuclear assemblies of 6 **(B)** Cell viability of iPSCs after incubating with 5, 7, or 9 for 2 h, **p < 0.01, ***p < 0.001 **(C)** Cell viability of iPS-derived HPCs, HS-5 cells, and HEK293 cells after incubating with 400 μM 5 for 2 h **(D)** Confocal laser scanning microscopy (CLSM) images of iPS cells after being treated by 5 (400uM) for 2 h. Copyright 2021, American Chemical Society.

Previous studies have shown that alkaline phosphatase (ALP) is overexpressed in a variety of malignant tumor cells. (Fishman et al., 1968). ALP dephosphorylation can removing phosphate groups from phosphorylated amino acid residues. Since dephosphorylation can greatly increases hydrophobicity, alkaline phosphatase is a common enzyme in the process of establishing enzyme-instructed self-assembly (EISA). Xu and co-workers conducted pioneering studies in EISA and Xu’s research group applied ALP to make supramolecular self-assembly involving the dephosphorylation of Fmoc-phospho-tyrosine firstly. (Yang et al., 2004). Recently, they found that ANL-phosphopentapeptide, catalyzed by ALP over-expressed by pluripotent stem cell (IPSCs), formed an intranuclear peptide assembly made of α-helices rapidly and selectively kills IPSCs (Figure 2A). (Liu et al., 2021) Confocal laser scanning imaging indicated that the L-pentapeptide assembly first self-assembles on the membrane after alkaline phosphatase dephosphorylation, forming a fluorescent spot, and then the assembly enters the cell and quickly enters the nucleus. And the aggregation may be related to cell death (Figure 2D). When the concentration decreased to 200μM, there was almost no fluorescence in the cells which indicated that self-assembly to form nanoparticles is essential for nuclear targeting. Using the L-phosphopentapeptide to treat iPS cells and normal cells separately, 93% of iPS cells were killed within 2 h (Figure 2B), while the L-phospho-pentapeptide treatment had an effect on normal cells (such as iPS differentiated hematopoietic progenitor cells (HPCs), HS-5 cells and HEK293 cells) had almost no effect (Figure 2C). And the L-phospho-pentapeptide and its assembly could be quickly hydrolyzed by the lysate of normal cells (HS-5), and would not have a long-term impact on normal cells. These results indicated that in a mixed cell population composed of IPSCs and non-IPSCs, IPSCs could be effectively and selectively eliminated by rapidly forming nanoribbons during the ALP-catalyzed dephosphorylation process. As the first case of intranuclear assembly of polypeptides, this work was not only clarified the application of enzymatic non-covalent synthesis in the selective targeting of the nucleus, but might also opened up a new way to eliminate other pathological cells that express certain enzymes. Yang’s group also achieved excellent studies in enzymatic self-assembly. Recently, they reported a new strategy, using enzymatic self-assembly method to prepare a series of artificial esterase with the same chemical composition but different catalytic performance, and measured their ability to catalyze hydrolysis. (Chen Y et al., 2021). In addition, they proposed a strategy for enzymatically self-assembling nanofibers to make fluorophores form excimer complexes at low concentrations and in biological environments. (Zhong et al., 2021). And they also used the strategy of combining small molecule peptides with EISA properties and AIE groups with high fluorescence brightness and reactive oxygen generation in the aggregated state, the identification and elimination of senescent cancer cells had been successfully achieved. (Gao et al., 2020). In another outstanding work, they constructed supramolecular nanostructures of dual anticancer drugs with synergistic effects. The resulting supramolecular could transport these two drugs to cells efficiently, especially to the cell nucleus. (Cai et al., 2017). These works showed that enzymatic self-assembly by ALP was relatively mature and could be an effective strategy for *in-situ* self-assembly.

#### 2.2.2 Cathepsin B

**FIGURE 3:**
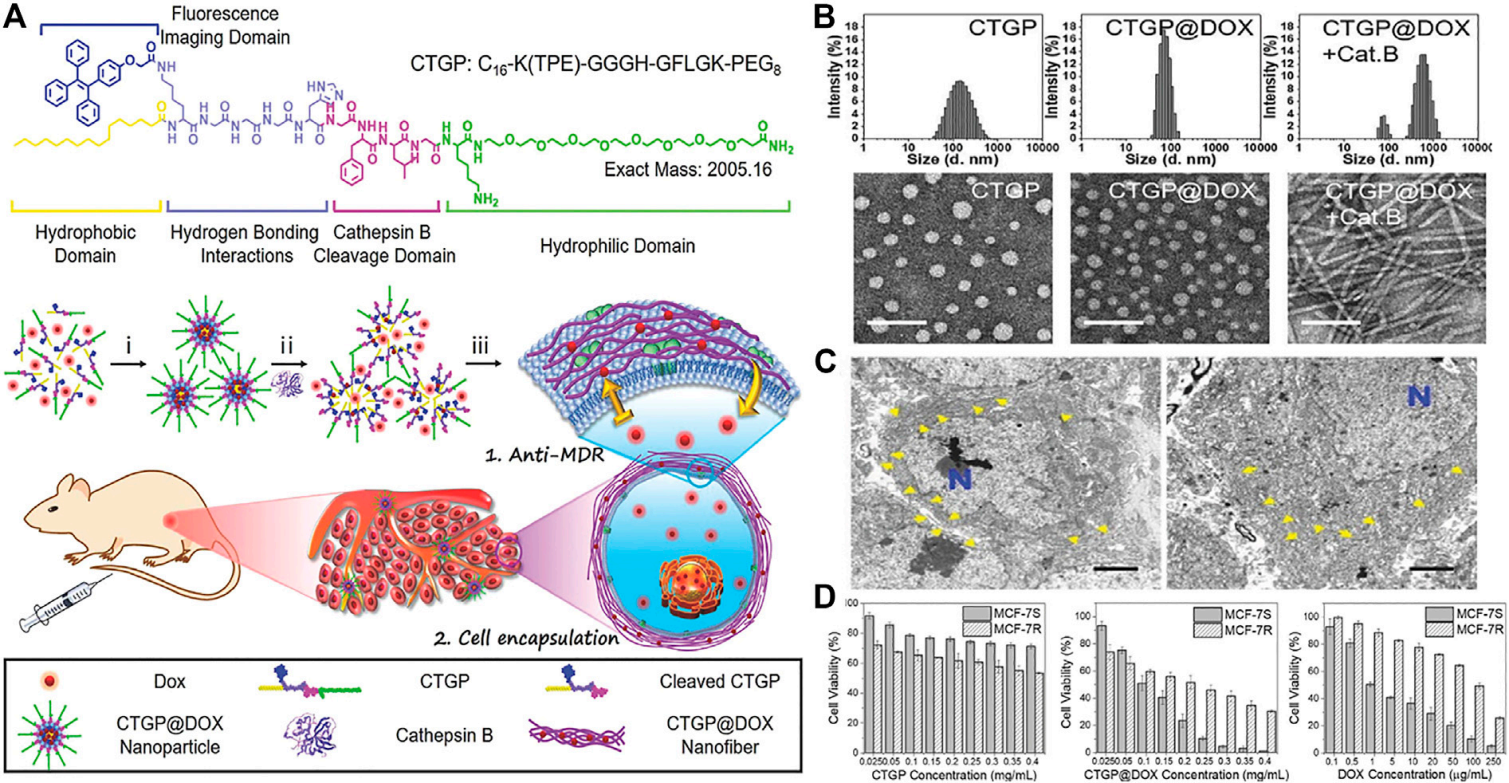
**(A)** The molecular structure of CTGP designed with the hydrophobic domain, fluorescence imaging domain, hydrogen bonding interactions domain, cathepsin B cleavage domain and hydrophilic domain. Proposed mechanisms of i) peptide-DOX self-assembly, ii) cathepsin B-instructed micelles disassociation with DOX release and fluorescence recovery, and iii) CTGP@DOX nanofiber reassembly on cell membrane for restricting DOX efflux and encapsulating cells **(B)** Hydrodynamic size and TEM images of CTGP nanomicelles, CTGP@DOX nanoparticles and CTGP@DOX treated with cathepsin B (donated as Cat. B). Scale bar: 100 nm **(C)** Bio-TEM images of MCF-7R cells treated with CTGP@DOX for 6 h (left) and 24 h (right), respectively. The blue “N” represents cell nuclei. CTGP@DOX nanofibers are highlighted by yellow arrows at pericellular space. Scale bar: 2 µm **(D)** Cytotoxicity of A CTGP, B CTGP@DOX, and C free DOX against MCF-7S and MCF-7R cells. Copyright 2018, Small.

Studies have shown that in some malignant tumors and precancerous lesions, the expression of cathepsin B (CTSB) is highly upregulated. And confirmed by genomic and proteomic analysis, many human cancers are associated with high expression of CTSB, such as esophageal cancer, glioblastoma, breast cancer and so on. Based on CTSB -catalyzed strategy, Zhang designed a transformable chimeric peptide to target and self-assembled on the cell membrane to encapsulate cells and overcome tumor multidrug resistance (MDR). (Zhang C et al., 2018). The chimeric peptide (C16-K(TPE)-GGGH-GFLGK-PEG8, denoted as CTGP) was composed of cathepsin B reactive Gly-Phe-Leu-Gly(GFLG) sequence, (Zhang et al., 2013; Yuan et al., 2015), hydrophilic polyethylene glycol (PEG) and Gly-His (GGH) sequence, cell membrane targeting hydrophobic 16 carbon alkane chain (Zhang et al., 2014; Tanaka et al., 2015) and aggregation-induced emission (AIE) probe. (Han et al., 2015). In the blood circulation process, CTGP@DOX can maintained stability with the PEG corona of CTGP, and further accumulate CTGP@DOX at the tumor site by enhancing its permeability and retention. Once CTGP@DOX encountered the high secretion of cathepsin B around tumor cells, it dissociated upon cathepsin B cleavage and reassembles into nanofibers on the cell membrane by targeting the hydrophobic 16-carbon alkyl chain through the cell membrane. These extracellular self-assembled nanofibers would greatly limit the outflow of DOX and effectively enhance its ability to resist MDR (Figure 3A). Both TEM image and DLS analysis showed that after CTGP@DOX was incubated with cathepsin B at 37°C for 24 h, the nanoparticles were transformed into nanofibers (Figure 3B). After 6 and 24 h incubation of MCF-7R cells, a large number of slender fiber-like structures can be seen on the plasma membrane. These results indicated that CTGP@DOX could be dissociated by cathepsin B, which was highly expressed by MCF-7S/R cells, and reassembled into nanofibers on the cell membrane, thereby achieving effective cell packaging (Figure 3C). They evaluated the cytotoxicity and anti-MDR of CTGP@DOX on MCF-7S/R cells by the methyl thiazolyl tetrazolium method and the apoptotic necrosis experiment. CTGP shows good biocompatibility and almost no cytotoxicity. At the same time, the median inhibitory concentration (IC_50_) of CTGP@DOX on MCF-7S cells and MCF-7R cells were 0.1 mg ml^−1^ and 0.2 mg ml^−1^, respectively. Therefore, the MDR factor of CTGP@DOX is 2 (0.2 mg ml^−1^/0.1 mg ml^−1^). For the free DOX group, the IC_50_ value of MCF-7S cells was about 1μg ml-1, and the IC_50_ value of MCF-7R cells was about 100 µgmL-1. The MDR factor of doxorubicin was 100 (100 μg ml^−1^/1 μg ml^−1^), which was 50 times that of CTGP@DOX, indicating that CTGP@DOX has an excellent anti-MDR ability (Figure 3D). In summary, they gave the first example of designing and applying cathepsin B response and cell membrane targeting amphiphilic chimeric peptide CTGP, demonstrating how peptide-based self-assembly and cell encapsulation could overcome tumor MDR. In addition, Wang also developed autocatalytic morphology *trans*-formation platform to improve tumor-specific drug accumulation by kinetic control. (Cheng et al., 2019b). In another work of Wang and co-workers, the autocatalytic growth strategy was first introduced into the CstB-catalyzed strategy to accumulate targeted drugs *in vivo.* (Cheng et al., 2019a).

#### 2.2.3 MMPs and Other Enzymes

**FIGURE 4:**
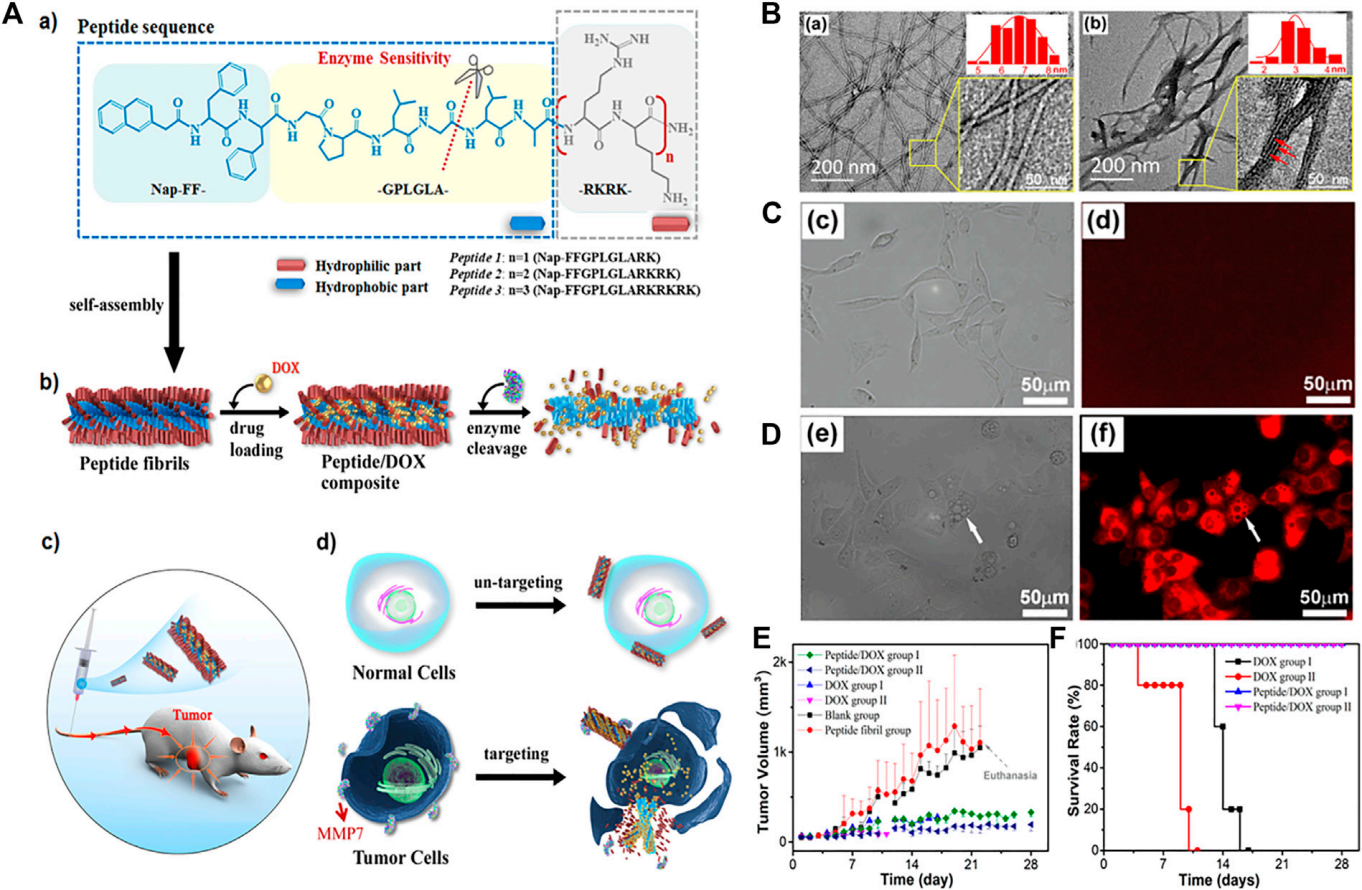
**(A)** (a) Molecular structures of designed MMP7-sensitive peptides and the expected enzymatic hydrolysis reaction. (b) Schemes showing the peptide self-assembled nanostructures and drug loading and enzymatic release processes. (c) Scheme illustrating the animal experiments for *in vivo* cancer therapeutic efficiency. (d) Schematic representation of cancer-targeted drug delivery and selective cancer killing. The drug-loaded fibrils retain their integrity at normal cell sites and do not release drug molecules. While at cancer cell sites the fibrils experience structural transition from well-dispersed thicker fibrils to thinner fibrillar structures and disassembled aggregates due to MMP7 hydrolysis, leading to targeted release and the accumulation of drug molecules around the cancer cells **(B)** TEM images of the self-assembled structures of peptide 2 at 0.25 mM before (a) and after (b) MMP7 treatment (insets) Magnified images with diameter distributions of the corresponding fibrillar structures. The size distribution in each case was calculated from at least 200 separate fibrils (b inset) Magnified image that shows the fibril bundles comprised of many thinner fibrillar structures. The thinner fibrillar structures are indicated by the red arrows **(C, D)** Microscopic images of COS7 cells (c, d) and HpeG2 cells (e–g) after coculturing with the peptide 2/DOX composites at 37°C and the nominal DOX concentration of 4.0 μM for 28 h (c and e) Bright field images (d and f) Fluorescence images **(E)** Variation of the tumor volume, survival ratio **(F)** Variation of the tumor volume, body weight. Copyright 2019, Acs Applied Materials and Interfaces.

In addition to phosphatase and cathepsin B, the enzymes of matrix metallopro-teinase (MMP), transglutaminase, caspase, gelatinase and so on are other illness related enzymes that were overexpressed and secreted by various disease cells. Wang’s group reported a near-infrared (NIR) peptide probe with MMP-2/9-induced self-assembly. (An et al., 2020). This peptide probe first specifically recognized the over-expressed α-v-β-3 integrin in renal cancer cells, and then was cleaved by the up-regulated MMP-2/9 in the microenvironment of tumor. The probe residues spontaneously self-assembled into nanofibers, which exhibited an excretion inhibitory effect in the kidney, could perform high-performance imaging of human renal cell carcinoma (RCC), and achieve complete tumor resection, and ultimately reduce postoperative recurrence. Lu and co-workers designed a supramolecular using MMP-7 sensitive peptide to encapsulate anticancer drug doxorubicin (DOX) for targeting tumor cells. (Cao et al., 2019). These peptides had three functional motifs: 1) NAP-Phe-Phe (NAP-FF). The aromatic segment promoted the self-assembly of the polypeptide in aqueous solution by providing a wide range of aromatic and hydrophobic interactions. 2) Gly-Pro-Leu-Gly-Leu-Ala (GPLGLA) was an enzyme substrate cleaved by MMP-7, which made the system enzyme sensitive. 3) (Arg-Lys)n((RK)n) promoted the interaction with the cell membrane. These peptides encapsulated a large amount of DOX to form fibrils, undergone morphological changes triggered by MMP7 in certain cancer cells, released drug molecules, and accumulated in the cancerous area to provide targeted delivery (Figure 4A). The peptide formed regular filaments in the buffer solution with a diameter of 7.0 ± 1.2 nm, however, after treatment with MMP7, the self-assembled nanostructure maintains the contour of the fibril, but was obviously thinner, with a diameter of only 3.0 ± 1.0 nm, which was easily form bundles through lateral union (Figure 4B). After treatment with the peptide 2/DOX complex, the microscopic images of COS7 and HpeG2 was taken. For COS7 cell, the bright field image showed that most of the cells are growing well, with a long spindle shape. But the fluorescence image was black in the entire view, indicating that the free DOX concentration in the cells was very low. (Figure 4C). For HpeG2, most of the cells were round and bright red, indicating that the cells had taken up a large amount of DOX (Figure 4D). The difference between normal cells and cancer cells was considered to be related to the difference in the expression level of MMP7. Subsequently, they conducted animal experiments to confirm the effect of the treatment *in vivo*. In the case of treatment with the supramolecular, tumor growth was also successfully inhibited. Compared with the blank control, the tumor volume growth rate slowed about 5–6 times (Figure 4E). What’s more exciting was that even for the higher dose of fibril-loaded DOX (12 mg/kg), the mice had a 100% survival rate and showed no significant changes in body weight throughout the experiment (Figure 4F). The results showed that the compound formulation could greatly increase the safe dose of DOX, improved the treatment efficiency, and significantly reduce its toxicity. In another work by Zheng and co-workers, (Lv et al., 2020), they designed RGD peptides to guide the self-assembly of monomeric recombinant proteins into nanoparticles, which provided insights for the design and development of integrin αvβ3 targeted protein nanoparticles for cancer treatment.

Moreover, recently, Zhong’s group designed a three-enzyme responsive self-assembling peptide derivative LND-1p-ES for carrier-free delivery of the drug Lonidamine (LND). (Wu et al., 2021). These nanofibers also promoted the controlled release of drugs with the help of cellular proteases, and showed enhanced efficacy and selectivity against melanoma cells A375 *in vitro* and *in vivo*. All in all, the above studies provided a feasible strategy to regulate *in-situ* self-assembly using enzymes overexpressed in diseased cells.

### 2.3 Redox Species

**FIGURE 5:**
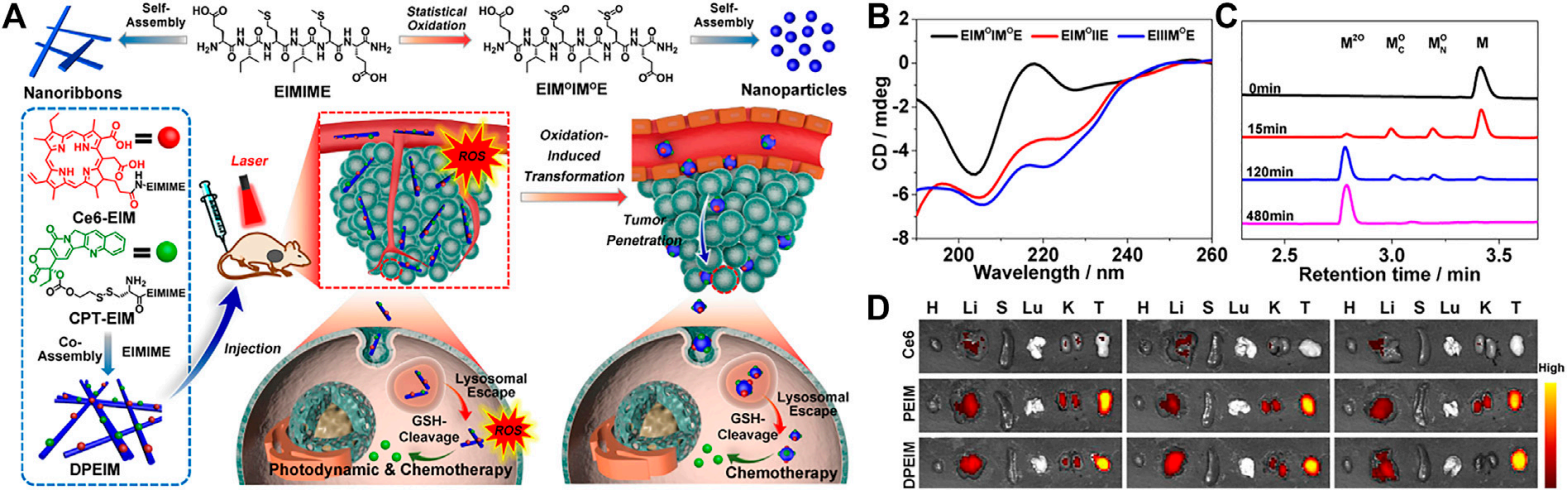
**(A)** Schematic illustration of creation of the peptide transformable scaffolds with cascade therapeutic effect controlled by *in situ* ROS generation. Top: Chemical structures of peptide EIMIME and its oxidized counterpart and their self-assembly into nanoribbons or nanoparticles, respectively. Bottom: Co-assembly of the hexapeptide with its derivatives Ce6-EIM and CPT-EIM into peptide scaffold DPEIM. The resulting scaffold underwent morphological transition promoted by *in situ* ROS generation, thus facilitating tumor penetration and enhancing combinatorial photodynamic and chemo-therapeutic efficacy **(B)** CD spectra of peptides EIMIMOE, EIMOIIE, and EIIIMOE **(C)** UPLC traces of peptide EIMIME (5 mM) oxidized by H_2_O_2_ at a concentration of 15 mM at different times **(D)** intravenously administrated with free Ce6, PEIM, or DPEIM at different time points. Quantitative mean fluorescence intensity of the *in vivo* signals at tumor sites in the mice. Copyright 2021, Nano Today.

The growth rate of tumor cells is significantly faster than that of normal cells, and their metabolism is also faster. Corresponding to this vigorous metabolism is that NADPH oxidase in the mitochondria and endoplasmic reticulum of tumor cells will produce a large amount of reactive oxygen species. (Tao and He, 2018). Yu and co-workers reported the self-assembly of oxidatively regulated peptides generated by *in-situ* ROS into a deformable scaffold for cascade cancer treatment. (Song et al., 2021). This ROS-responsive morphology transformable scaffold DPEIM consisted of three parts, hexapeptide EIMIME, photosensitizer chlorin e6 (Ce6) (Ce6-EIM) and chemotherapeutics camptothecin (CPT-EIM). Polypeptide oxidation caused by *in-situ* generated intracellular or extracellular ROS promoted the morphological transformation of the polypeptide scaffold, thereby promoting tumor penetration. The polypeptide scaffold DPEIM was internalized before being oxidized by the tumor cells at the edge of the tumor tissue. The production of intracellular ROS under laser irradiation contributed to the photodynamic therapy of DPEIM, and it was accompanied by chemotherapy produced by the release of CPT induced by GSH (Figure 5A). They used hydrogen peroxide (H_2_O_2_) as an external oxidant to study the conformation of the oxidized peptide and the morphology of the nanostructure formed by the oxidized peptide. Under physiological conditions, adding three equivalents of H_2_O_2_ to the peptide solution could quantitatively convert sulfide into sulfoxide (Figure 5B). After the DPBF probe was depleted, the oxidation of methionine residues in UV/VIS samples of DPEIM and PEIM was estimated. The UPLC profile clearly showed that all the peptide components in the UV/Vis samples of PEIM and DPEIM are quantitatively converted into oxidized peptide components, which indicated that the *in-situ* generated ROS induced the oxidation of methionine in the peptide. In particular, no oxidation intermediate containing a sulfoxide group was detected, which was consistent with ^1^O_2_ having a stronger oxidation ability than H_2_O_2_ (Figure 5C). Both *in vivo* and *in vitro* results showed that the conformational transition of the polypeptide EIMIME and the morphological transition of the polypeptide EIMIME assembly induced by the oxidation of methionine residues prove the establishment of an oxidation-regulated self-assembly platform. They further studied the biodistribution of peptide scaffolds in the main organs and tumor tissues of 4T1 breast tumor-bearing mice (Figure 5D). The results confirmed that compared with the free therapeutic drug alone, the accumulation and retention time of the therapeutic drug in the peptide stent at the tumor site were improved, thus proving the role of the peptide stent in effective drug delivery. In another work, Gao and co-workers developed a redox-responsive supramolecular assembly through oxidation elimination reaction. (Wei et al., 2018). After oxidized by H_2_O_2_, the formation of intramolecular hydrogen bonds produced fluorophore BQH, which leaded to molecular flattening, promotes intermolecular π-π stacking, and promoted self-assembly. Moreover, the assembly with fluorescent BQH18 molecules could easily distinguish cancer cells from normal cells.

**FIGURE 6:**
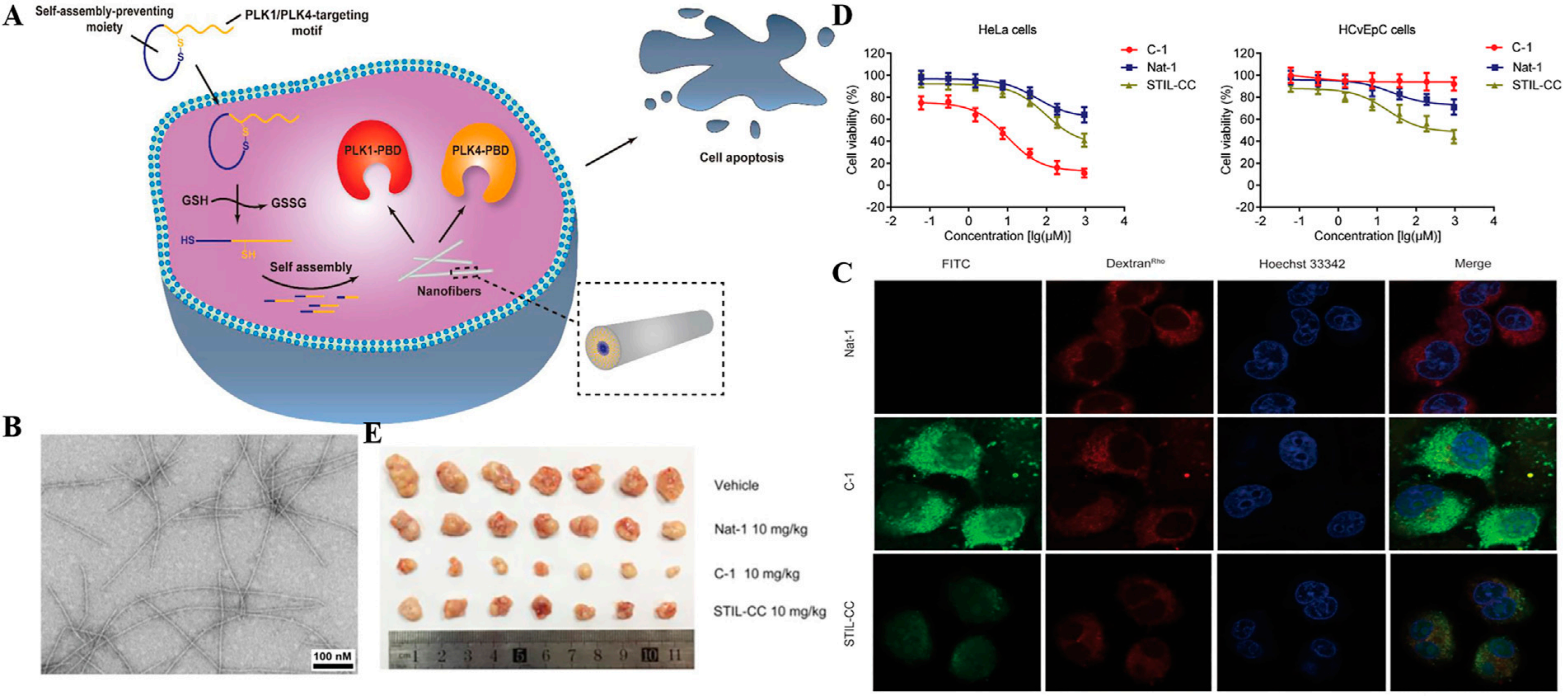
**(A)** Schematic illustration for cancer cell death induced by a redox-responsive bispecific PLK1/PLK4 nanomedicine derived from cyclic peptide precursor, termed as C-1 **(B)** TEM images of the fibers derived from the C-1 solutions (2 wt%) after the addition of GSH (10 × 10^–3 m^) at pH 7.6 (scale bar = 100 nm) (C) Confocal laser scanning microscopy images of HeLa cells cultured with 1 × 10–6 m of FITC-labeled Nat-1 (top panel), C-1 (middle panel), and STIL-CC (bottom panel) for 2 h. Rhodamine-labeled dextran (dextranRho) is an endocytosis marker. Nucleus was stained with Hoechst 33342 **(D)** Effect of C-1, Nat-1, and STIL-CC on a) HeLa cell and b) HCvEpC cell proliferation as determined by MTT assay. Data reported represent the mean ± SD from three independent experiments **(E)** *In vivo* antitumor activities of C-1. The image of tumors. Copyright 2020, Advanced Functional Materials.

Cells tend to maintain redox homeostasis and therefore also highly express reduced glutathione(GSH). (Yang et al., 2013). Niu and co-workers reported a redox-responsive bifunctional supramolecular nanomedicine based on self-assembly of cyclic peptides, named C-1, targeting PLK1 and PlK4 as an effective anticancer agent. (Yang DS et al., 2020). In this work, an effective PLK1-PBD inhibitor called linear peptide 4 was identified for the first time. In order to modify the linear polypeptide 4 into a specific nanomedicine in response to a thiol reducing agent such as GSH, the cyclic peptide precursor C-1 was constructed. When entering the cytoplasm of cancer cells, the high concentration of glutathione in the cell reduced the connection of disulfide bonds, causing it to form a linear conformation, and self-assembled into nanofibers (Figure 6A). They observed the self-assembly behavior of C-1 *in vitro* through transmission electron microscopy (TEM). The results confirmed that GSH can convert C-1 into linear peptides (called L-1) and lead to the formation of nanofibers but TEM images of C-1 solution show no obvious fibers without GSH (Figure 6B). Afterwards, they explored the characteristics and feasibility of the bispecific precursor C-1 for intra-cervical cancer imaging. The cytoplasm and nucleus of HeLa cells treated with FITC-C-1 had strong green fluorescence signals, indicating that it can effectively escape from the cell body to the cytoplasm and nucleus (Figure 6C). In addition, they further studied the selectivity of C-1 to inhibit the proliferation of HeLa cells and HCvEpC cells, and the results showed that C-1 has a selective inhibitory effect on the proliferation of cervical cancer cells (Figure 6D). At the same time, *in vivo* experiments also confirmed C-1 significantly inhibited cells-induced xenograft tumor growth (Figure 6E). In another example by the Ding group, (Xu et al., 2018), a new type of curcumin supramolecular nanofibers (Cur-SNF) were prepared through self-assembling short peptides in the response of GSH, which greatly improved the sensitivity of colorectal cancer to ionizing radiation.

### 2.4 Tandem Assembly by the Combination of Two Stimulus

**FIGURE 7:**
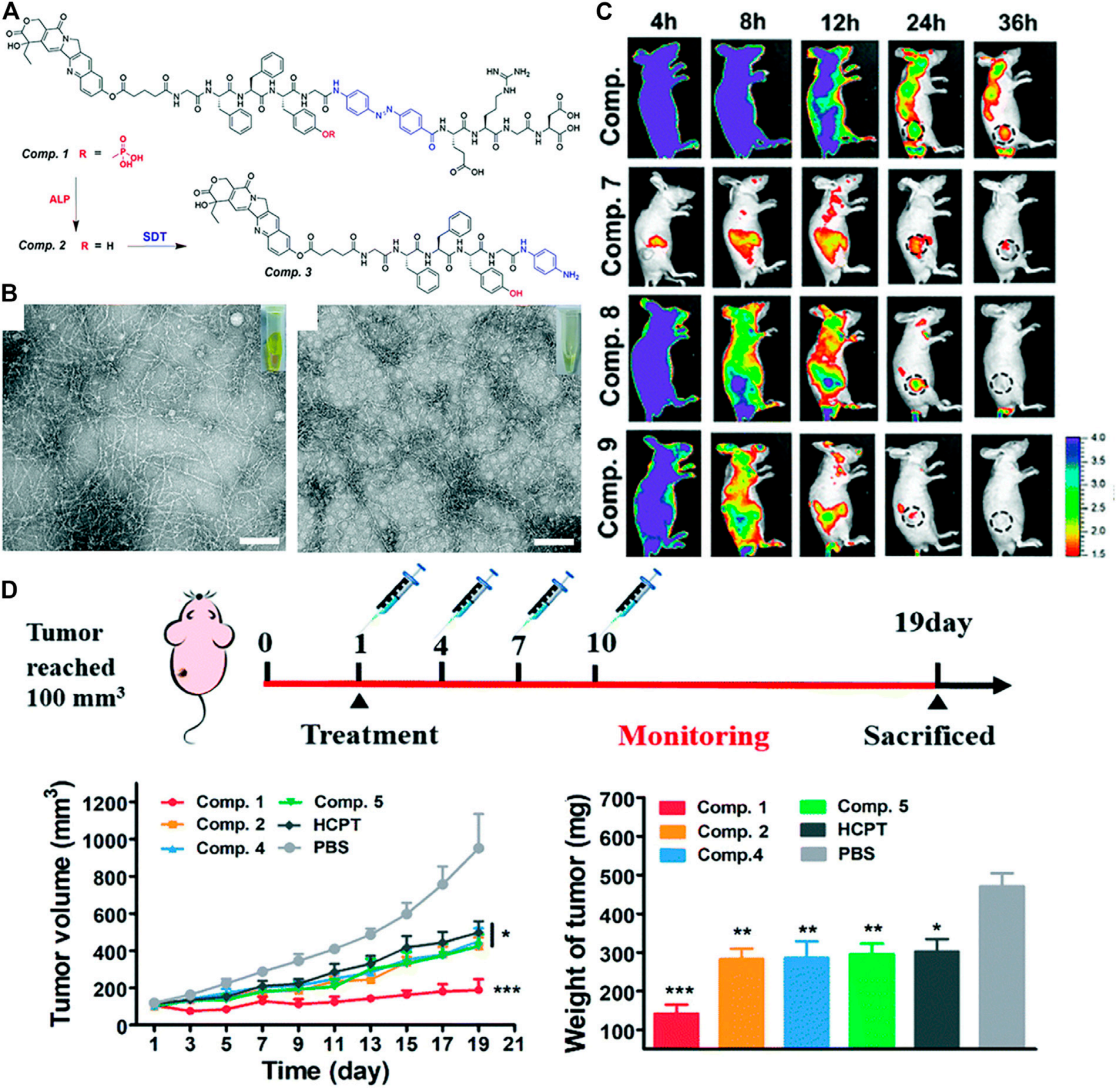
**(A)** Enzyme instructed chemical structure conversions from Comp. 1 to 2 by ALP and then Comp. 2 to 3 by SDT **(B)** TEM and optical images of Comp. 2 acquired by ALP catalysis and (left) Comp. Three acquired by SDT catalysis, scale bar = 100 μm (right) **(C)** Representative pictures of NIR-fluorescence imaging of A549 tumor bearing nude mice with the treatment of Comp. 6, 7, 8 and 9 at different time points after the intravenous administration **(D)** Treatment schedule for inhibition of A549 tumors in BALB/c nude mice. The treatment of A549 tumor bearing nude mice by the intravenous injection of Comp. 1, 2, 4, 5, HCPT and PBS at a dose equaled 3 mg kg^−1^ equivalent of HCPT (top). Tumor volume (bottom, left) Tumor weight (bottom, right). Copyright 2021, Nanoscale.

The locally one-step self-assembling of prodrugs into nanomedicine have been studied extensively. Nevertheless, the degradation and disassembly of nanomedicine after self-assembly impeded the improvement of efficiency. It is still challenging to prevent such unwanted disassembly and biodegradation. To address the problem, tandem assembly by connecting two steps of self-assembling was developed. (Tang et al., 2018; Zhan et al., 2018; Zheng et al., 2019). Recently, Yang and co-workers designed a novel drug delivery strategy based on tandem SA (self-assembly), by self-assembling 10-hydroxycamptothecin (HCPT) *in-situ* at the tumor site, and then prevented its decomposition and biodegradation in cancer cells through tandem SA, specifically delivering HCPT to lung cancer. (Zheng et al., 2021). In this study, they conjugated HCPT with peptides. The obtained molecule HCPT–GFFpYG–N=N–ERGD (Figure 7A, compound 1) was a precursor with tandem self-assembly capability. The precursor could be converted a fibrous network *via* by alkaline phosphatase catalysis (Figure 7A, compound 2). The generated nanofibers were catalyzed by the intracellular reductase to form more hydrophobic nanofibers (Figure 7A, compound 3), preventing its decomposition and biodegradation, and further improving the anti-lung cancer effect of HCPT. The self-assembly behavior of tandem molecules of compound1 catalyzed by ALP and reductase was demonstrated by TEM (Figure 7B). In the distribution experiment of tandem self-assembled molecules in A549 tumor-bearing BALB/c nude mice, the fluorescent signal of the fluorescent molecule of compound 1 at the tumor site could last up to 36 h. The results showed that the tandem molecular SA strategy could improve the accumulation, penetration and retention of prodrugs. they evaluated the therapeutic potential of tandem self-assembled prodrugs for solid tumors in a BALB/c nude mouse model bearing A549 cells (Figure 7C). Furthermore, the therapeutic potential of tandem self-assembled prodrugs on BALB/c nude mouse model solid tumors bearing A549 cells was evaluated. Surprisingly, after the first administration of compound 1, the tumor volume began to decrease and tumor volume just increased slightly at the 19th day (Figure 7D).

### 2.5 Other Therapy Besides Directly Drug Delivery

**FIGURE 8:**
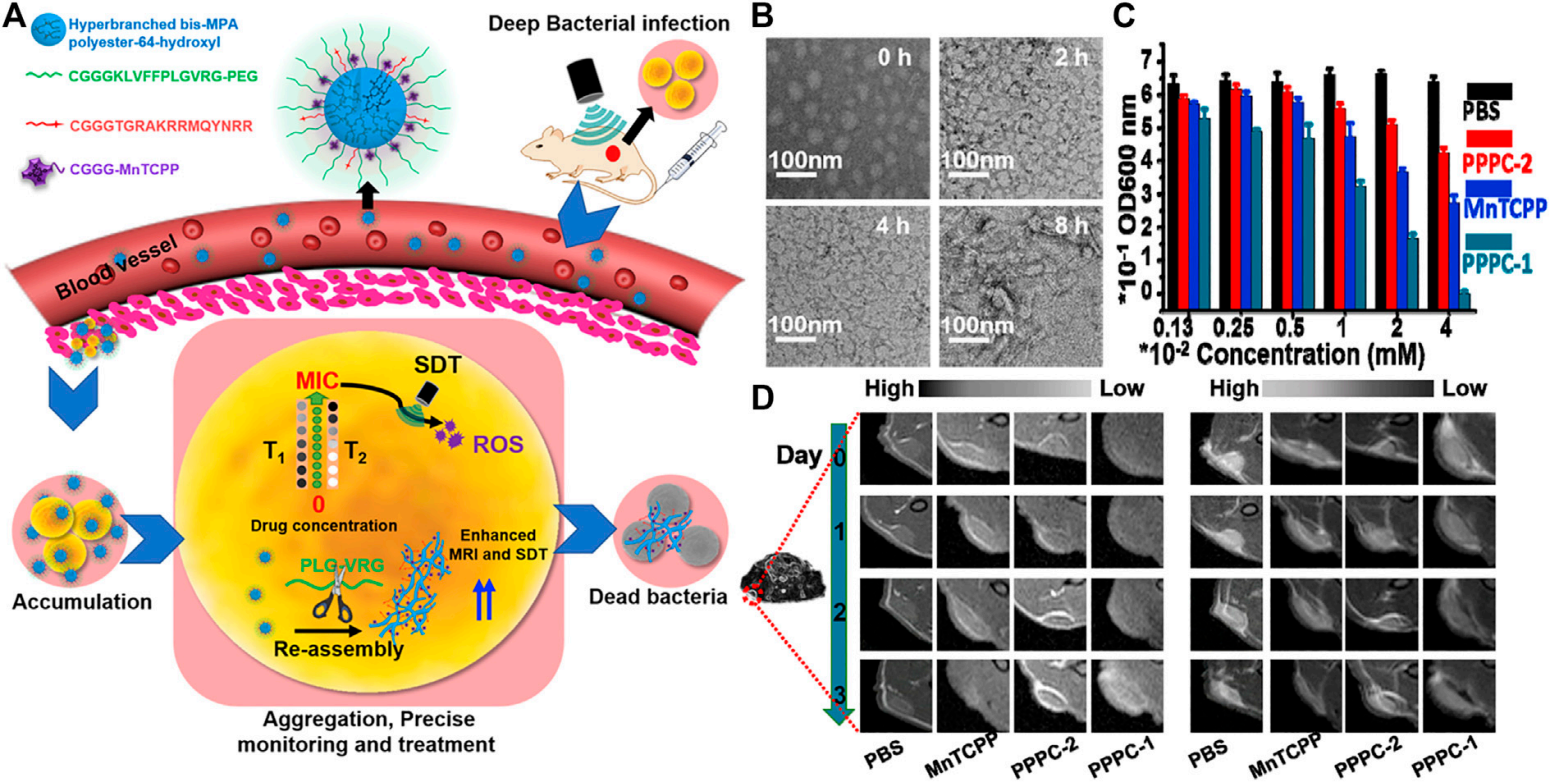
**(A)** Schematic illustration of enzyme-induced morphology transformation of PPPC for precise magnetic resonance imaging-guided treatment of drug-resistant bacterial deep infection. Hyperbranched bis-MPA polyester-64-hydroxyl as backbone can be linked with other parts after modification, and the relative hydrophobicity of backbone can self-assembled in the “inner region”. CGGGKLVFFPLGVRG-PEG is a self-assembled peptide (KLVFF) linked with an enzyme-cleavable peptide (PLGVRG) and the PEG terminal, CGGGTGRAKRRMQYNRR is a bacterial targeting peptide, and CGGGMnTCPP is the sonosensitizer (MnTCPP) segment **(B)** TEM of PPPC-1 that were dispersed in gelatinase (10 μg/ml) Tris buffer solution (pH 7.4) at various times **(C)** Antibacterial properties of PPPC-1, PPPC-2 and MnTCPP molecule toward *S. aureus* under US irradiation (1.0 MHz, 1.5 W/cm^2^) for 9 min at different concentrations (CMnTCPP) **(D)** Representative MRI T1 contrast in bacterial infection site (red circle) in mice injected by PBS, MnTCPP, PPPC-2, and PPPC-1 for 3 days (left) Representative MRI T2 contrast in bacterial infection site (red circle) in mice injected by PBS, MnTCPP, PPPC-2, and PPPC-1 for 3 days (right). Copyright 2021, Biomaterials.

In addition to the treatment of cancer, supramolecular peptides are also used in the therapy of myocardial infarction, (Chen R et al., 2021), osteoarthritis, (Kim et al., 2016), ocular inflammation, (Yu et al., 2018), antibacterial and biomimetic platelets. (Yang PP et al., 2020). It is worth mentioning that antibacterial is one of the most widely used. As early as 2012, Zhang et al. designed a new AMP KRRFFRRK (named FF8) antimicrobial peptide. (Shen et al., 2020). It specifically targeted the negatively charged bacterial membrane and self-assembled into nanofibers on it. The tension generated by the formation of polypeptide nanofibers destroyed the lipid membrane, causing the membrane to rupture, and greatly improving its antibacterial activity. Subsequently, in 2014, Chin and co-workers combined ultraviolet with self-assembling peptides supramolecular. (Reithofer et al., 2014). An antibacterial hydrogel containing silver nanoparticles was prepared using ultrashort self-assembling peptides and silver nitrate. Under ultraviolet light, the silver nitrate could convert into silver nanoparticles easily. Then, in 2019, Li et al. reported a pH-adjustable antibacterial hydrogel with nanofiber network based on the design of octapeptide (IKFQFHFD) self-assembled under neutral pH conditions, which was used to remove biofilms and save chronic wound healing delays. (Wang J et al., 2019). And recently, in one of Wang‘s work, based on the “*in vivo* self-assembly” strategy, a polymer-peptide-porphyrin conjugate (PPPC) that could be monitored at the site of infection in real time was developed for precise deep sonodynamic therapy (SDT). (Wang D et al., 2021). PPPC was composed of four parts, hyperbranched polymer backbone, gelatinase-responsive self-assembling peptide, bacterial targeting peptide and porphyrin sonosensitizer (MnTCPP) fragment. Once the gelatinase-responsive PPPC nanoparticles (PPPC-1) reached the infected area, the hydrophilic PEG was removed and the PPPC reassembled, which enhanced the accumulation of sonosensitizers and reduced the minimum inhibitory concentration (MIC), so that they could achieve precise and efficient sterilization under ultrasonic irradiation (Figure 8A). PPPC could self-assemble in aqueous solution. Transmission electron microscopy (TEM) results showed that PPPC-1 assembled into spherical nanoparticles in the presence of gelatinase (Figure 8B). *Staphylococcus aureus* was co-cultured with the transformable PPPC (PPPC-1), morphology-unchanging analogues (PPPC-2) and MnTCPP in PBS, and then ultrasonic irradiation was applied to different time scales. The turbidimetric method was used to determine the acoustic toxicity of PPPC to bacterial cells, thereby evaluating the influence of the morphological changes of PPPC on the antibacterial effect of SDT *in vitro*. The results showed that PPPC-1 with a C_MnTCPP_ = 0.04 mM can completely inhibit the bacteria when the ultrasound time reaches 9 min. In contrast, the inhibition rates of PPPC-2 and MnTCPP on bacteria were ∼35 and ∼60%, respectively (Figure 8C). The transformable PPPC-1 had stronger antibacterial ability, which might be due to the fact that after the polyethylene glycol shell was shed off under the condition of gelatinase overexpression, the bacterial targeting peptide enhanced the interaction with the bacterial membrane, and subsequently Secondary assembly. Then PPPC-1, PPPC-2 and MnTCPP molecules were injected into BALB/c mice to study precise antibacterial *in vivo* guided by MRI. The results showed that for the PPPC group, the concentration calculated based on the T1 value is similar to the concentration calculated based on the T2 value, but for the MnTCPP group, the concentration calculated based on the T1 value and the T2 value was significantly different to calculate the concentration of each group at the target site, which indicated that MRI can accurately quantify MnTCPP in PPPC instead of free MnTCPP (Figure 8D).

**FIGURE 9:**
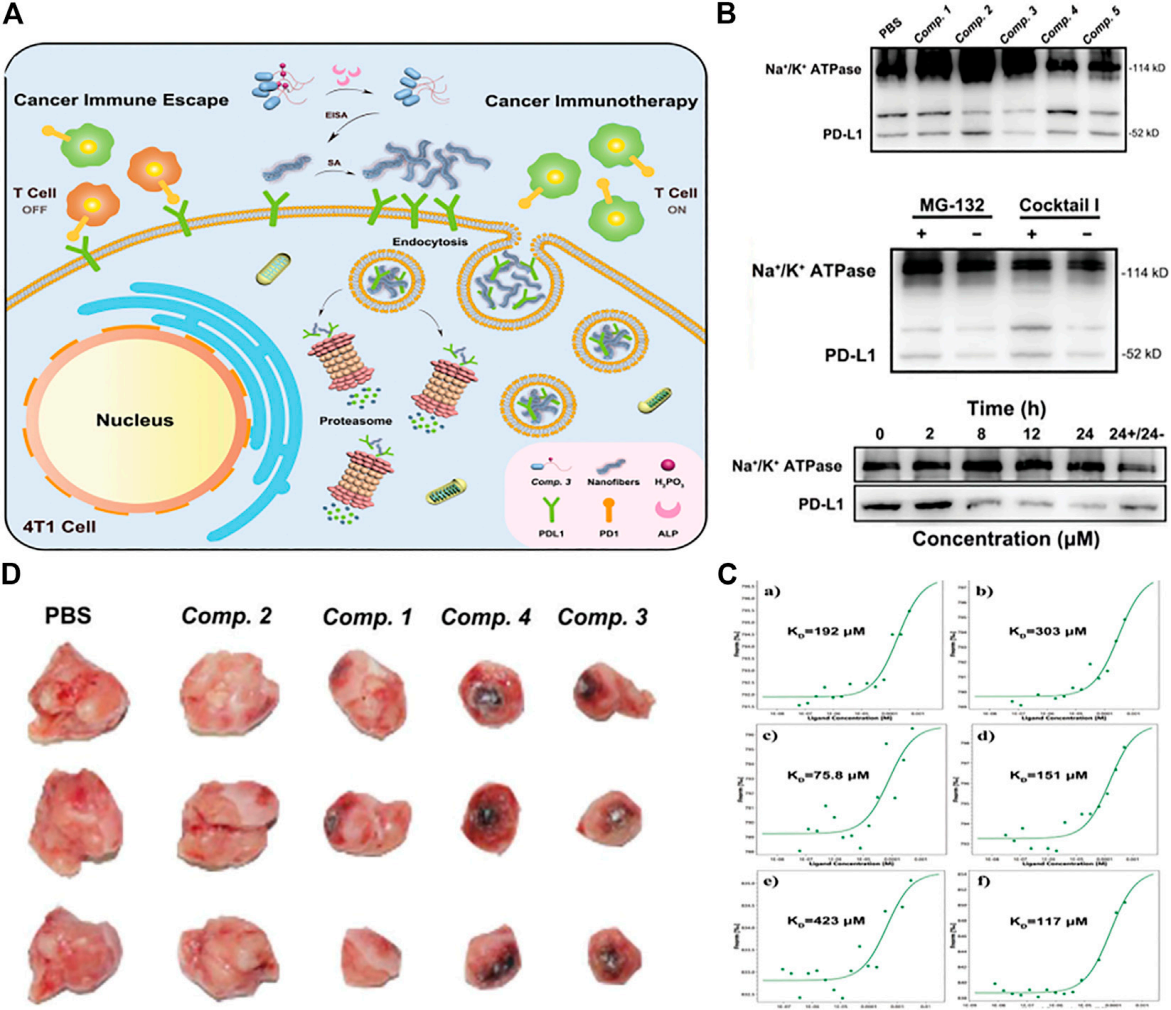
**(A)** Schematic explaining the degradation of membrane PD-L1 in 4T1 cells by ALP-catalyzed and PD-L1-guided peptide self-assembly of Comp. 3 **(B)** (top) cell membrane of 4T1 cells treated with Comp. 1–5 (100 µM) for 24 h (middle)with and without pretreatment of proteasome inhibitor MG-132 and ALP inhibitor Cocktail I and then with Comp. 3 (100 × 10–6 m) for 24 h. Western blotting of PD-L1 and Na^+^/K^+^ ATPase expressed on the cell membrane of (bottom) 4T1 cells treated with Comp. 3 (100 µM) for different times (the rightmost sample was treated with Comp. Three for 24 h and then with Comp. 3-free medium for another 24 h) **(C)** Microscale thermophoresis results of a–f) Comp. one to five and DPPA-1 to mPD-L1 at different concentrations. Their KD values are shown in the corresponding curves **(D)** Optical images of 4T1 tumors isolated from mice sacrificed by tail vein administration of Comp. 1–4. Copyright 2021, Advanced Functional Materials.

Due to the similar structural features to natural proteins, peptide molecules were expected to solve these challenges by directly triggering an immune response or improving drug delivery effects. (Li et al., 2019; Demircan et al., 2020). According to the mechanism of immune activation, therapeutic agents for different immune responses could be divided into emerging categories such as cancer vaccines, immune adjuvants, cytokines, checkpoint blocking, and engineered T cells. Currently, checkpoint blocking immunotherapy was a promising strategy for clinical trials. (Byun et al., 2017; Ribas and Wolchok, 2018). In view of this, recently, Yang group reported a new strategy to selectively degrade programmed cell death ligand 1 (PD-L1) membrane protein in cancer cells. (Wang Y et al., 2021). They combined a high binding affinity to PD-L1 with a phosphorylated peptide to make a precursor of a self-assembling molecule, which is compound.3 of sequence Ada-G^D^F^D^FpY^D^N^D^Y^D^S^D^K^D^P^D^T^D^D^D^ R^D^Q^D^Y^D^H^D^F. Extracellular alkaline phosphatase (ALP) could effectively catalyze the dephosphoryl -ation of compound.3. The overexpressed PD-L1 on tumor cell membrane provided binding sites for peptide supramolecular. These factors leaded peptide derivatives to form nanoparticles around PD-L1 on the cell membrane. Then, self-assembled Compound.3 combined with PD-L1 to simulate the partial denaturation state of PD-L1, therefore isolated its function. The peptide-PD-L1 complex was then taken up by the cell into the cytoplasm and further degraded through the proteasome degradation pathway (Figure 9A). In western blotting experiments, compound.3 has the most significant knockout effect on PD-L1 on the cell membrane of 4T1 cells, but this effect was not observed in normal cell line LO2 cells. Co-incubation of compound.3 and proteasome inhibitor MG-132 could slightly restore the level of PD-L1 on the cell membrane, and moderate accumulation of PD-L1 in the cytoplasm indicated that proteasome was one of the degradation pathways *in vivo*. The remarkable finding was that with the extension of the compound.3 incubation time, the level of PD-L1 on the cell membrane gradually decreases, however, replaced the culture medium containing compound.3 with fresh culture medium, and continued incubating for 24 h, the content of PD-L1 on the cell membrane would recover to a certain extent (Figure 9B). Micro thermophoresis (MST) was used to study the binding affinity of the material, which is obtained by treating compound one to four, with ALP and DPPA-1 in the extracellular domain of mouse PD-L1 (mPD-L1). The results showed that the binding affinity of DPPA-1 to mPD-L1 was improved through molecular self-assembly and the samples in compound one to four reacted with ALP overnight. The material obtained by treating compound.3 with ALP showed the highest binding affinity to mPD-L1, which might be attributed to the multivalent binding sites displayed on the surface of the enzyme self-assembly, indicating that it has great potential to manipulate PD-L1 levels (Figure 9C). Then they evaluated the anti-tumor effect of compound one to four in the BALB/c mouse 4T1 cell xenograft model. The tumor volume decreased by 23.7% (of the initial volume) in compound 3 groups compared with the PBS group (Figure 9D), which showed that compound 3 still showed the most outstanding anti-tumor effect.

## 3 *In-situ* SA Peptides for Diagnosis

### 3.1 Detection

**FIGURE 10:**
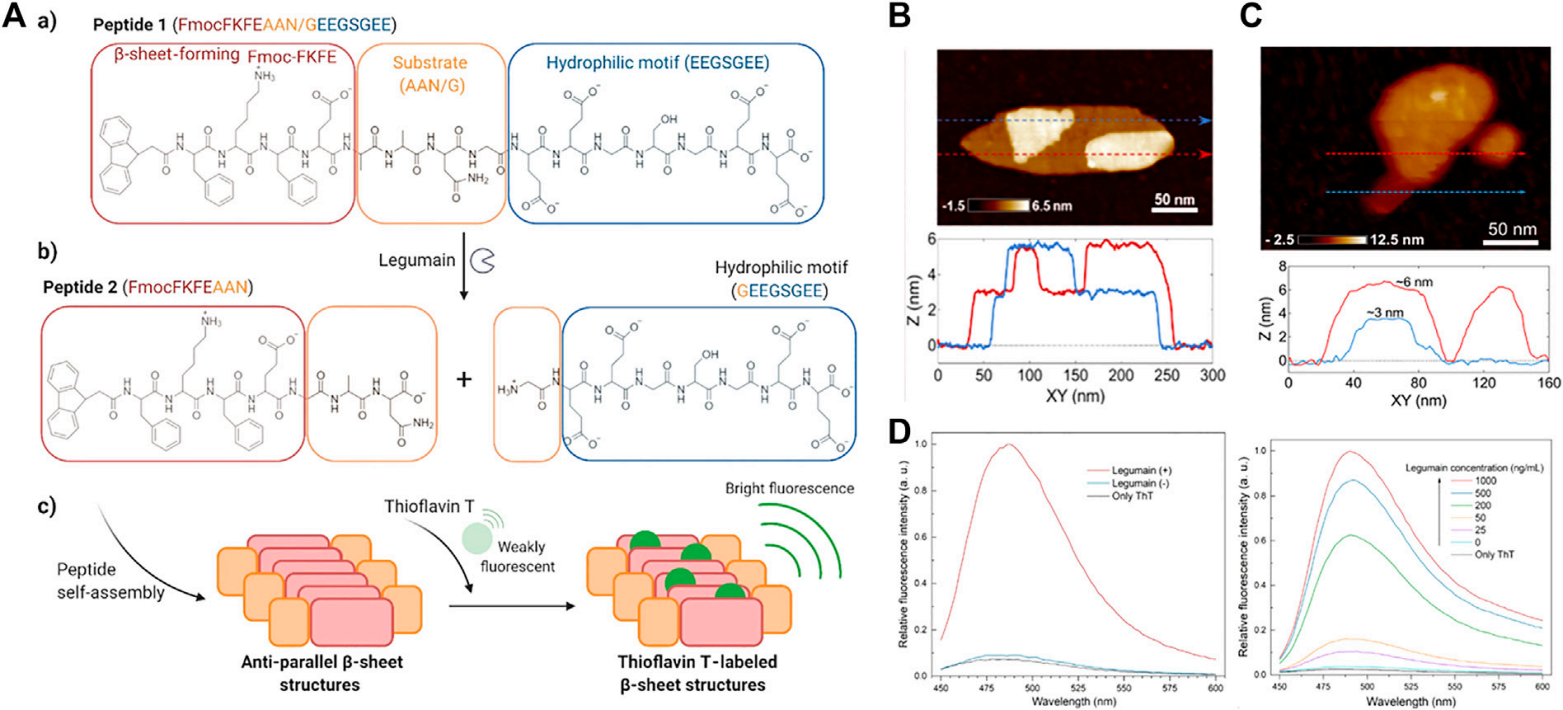
**(A)** ^a^Molecular structures of peptide 1 (a) and peptide 2 (b) formed upon hydrolysis of peptide 1 by legumain. (c) Schematic showing the self-assembly of peptide 2 and ThT labeling of the β-sheet structures **(B)** shows a high-resolution image of a plate-like structure and two individual thickness profile measurements (see the blue and red lines) **(C)** AFM characterization of peptide 1 incubated with 1,000 ng/ml legumain at 37°C for 2 h. Sequentially acquired AFM images of a nanoplatelet show excavation of the layered peptide material using the AFM probe (top). Height measurements corresponding to the dashed arrows in the AFM images show that the observed structures are composed of layers that are approximately 3 nm in thickness (bottom) **(D)** Representative fluorescence spectra of ThT (25 μM) in 10% plasma in the presence or absence of peptide 1 (1 mg/ml) and after 2 h incubation with or without legumain (1,000 ng/ml) (Left) Representative fluorescence spectra of ThT (90 μM) in the presence or absence of peptide 1 (1 mg/ml) and after 2 h incubation with different amounts of legumain (right). Copyright 2021, ACS Applied Nano Materials.

Polypeptides have shown potential in drug release, bioimaging and tissue engineering, (Zhang et al., 2012; Eren Cimenci et al., 2019; Sever-Bahcekapili et al., 2021), because their assembly can produce a variety of specific nanostructures and multi-functions, with high biocompatibility. Self-assembling peptide supramolecules have also gained great interest in detection applications. For example, CdS quantum dots coated with peptide-functionalized nanowires were used to detect mercury ions (Hg^2+^) in aqueous solutions rapidly, sensitively and selectively. (Meng et al., 2016). The use of peptide self-assembly also provided new opportunities for designing more sensitive molecular probes for detecting protease activity. For instance, in a work by Haam, they constructed an intelligent and effective tumor metastasis-related biomarker targeting nanoparticle system, calcein-loaded PeptiSomes, and used it for the quantitative detection of tumor metastasis and invasion-related proteases. (Kim et al., 2017). In another work by Wang, the biotinylated Asp-Glu-Val-Asp (DEVD) peptide substrate was immobilized on the surface of the gold electrode as a specific cleavage site for caspase-3. The strong interaction between biotin and the streptavidin linker triggered the formation of a biotin-FNP network on the electrode surface, thereby significantly increased the electron transfer resistance of the electrode. But, when the peptide substrate was cleaved by caspase-3, the biotin label would fall off the sensor surface, which prevented the capture of SA and the formation of the biotin-FNP network. This method had been successfully used to detect the activity of caspase-3 in HeLa cells treated with different anticancer agents. (Xia et al., 2020). Recently, Yildirim and co-workers developed a simple and sensitive method to monitor specific protease activity in biological solutions. (Olson et al., 2021). The β-sheet molecular probe that was hydrolyzed by the protease of interest was composed of three modules: 1) β-sheet development-like motif, 2) protease substrate, and 3) dissolution without protease activity Probe and prevent its self-assembly hydrophilic phantom. The substrate was cleaved by the corresponding protease to release the hydrophilic domain and trigger the formation of β-rich, 3 nm-thick self-assembled nanoplatelets (Figure 10A). The self-assembled nanostructure formed by peptide 2 was characterized by transmission electron microscopy and atomic force microscopy (Figure 10B). The results showed that platelets form micron-sized aggregates, ranging in size from tens of nanometers to hundreds of nanometers, and similar to peptide 2, the transmission electron microscope image of peptide 1 formed after being cleaved by legume protease showed a similar nanoplatelet structure. The AFM measurement further verified the formation of nanoplatelets with a thickness similar to that observed with peptide 2 (Figure 10C). These results indicated that peptide 2 molecules produced after peptide 1 was hydrolyzed by legume protease form nanoplatelets. Subsequently, peptide 1 and THT were used to determine legumain protease activity. Peptide 1 was incubated with different amounts of legumain (10–1,000 ng/ml) for 2 h, and thioflavin T (Th T, 90 μM) was added. As the concentration of legumain increased, the luminous intensity of THT gradually increased (Figure 10D). Finally, the legumain activity in human plasma was detected. Under optimized analysis conditions, in 10% plasma, when the concentration of legumain was 1,000 ng/ml, the fluorescence increase was about 20 times (Figure 10E). Surprisingly, the method described here might also be applicable to a variety of other applications, formed enzyme-triggered hydrogels to *in vivo* imaging of protease activity.

### 3.2 Imaging

**FIGURE 11:**
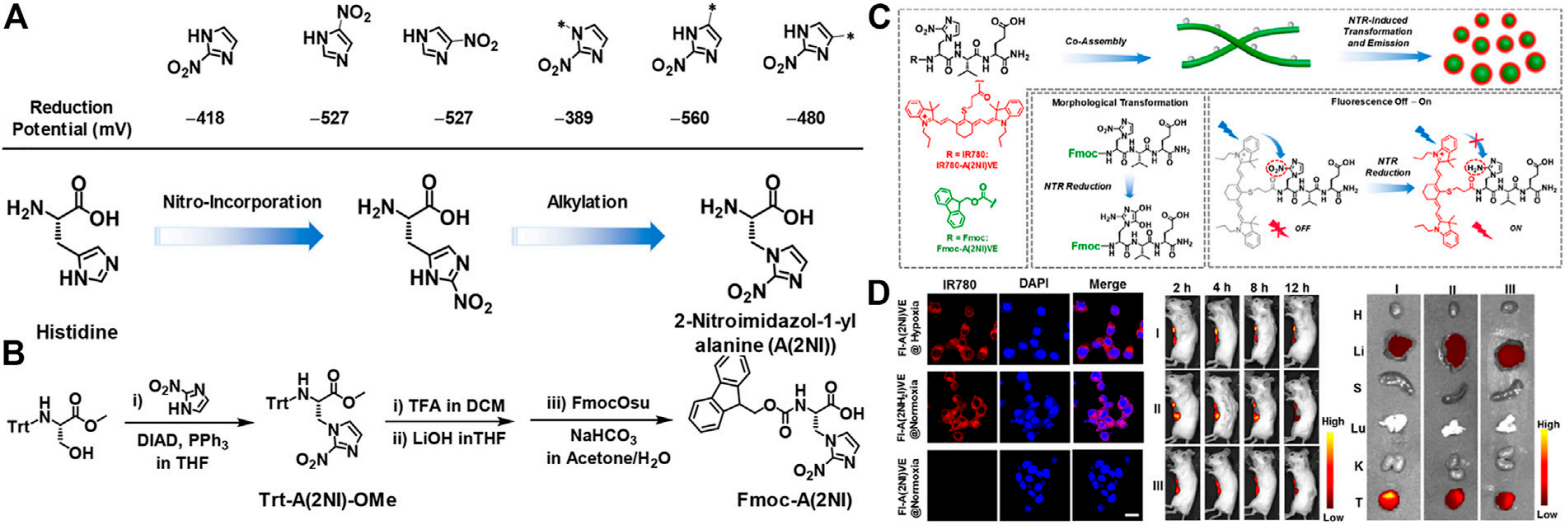
**(A)** (Top) The reduction potential (vs standard H-electrode) of nitroimidazole units associated with the position of the nitro-group or the alkylation (methylation) of 2-nitroimidazole (Bottom) The route for design of A(2NI) starting from histidine *via* considering the positions for incorporation of the nitro group and the alkylation of 2-nitroimidazole **(B)** Synthetic route for Trt-A(2NI)-OMe and Fmoc-A(2NI) **(C)** Schematic representation of the morphology-transformable supramolecular probes *via* coassembling Fmoc-A(2NI)VE and IR780-A(2NI)VE, in which NTR-reduction of A(2NI) in Fmoc-A(2NI)VE and IR780-A(2NI)VE lead to the morphological transition of the nanostructures and recovery of the emission of IR780 moieties, respectively. The gray dots along the nanofibers and the red circles around the particles denote the quenched and luminescent IR780 moieties, respectively **(D)** CLSM images of breast tumor cell 4T1 treated with FI-A(2NI)VE under the hypoxic or normoxic condition or FI-A(2NH2I)VE under normoxic condition. Scale bar: 20 μm (left) *In vivo* or *ex vivo* fluorescence imaging of 4T1 breast tumor-bearing mice (middle) or the tumor tissues and major organs dissected from the mice at 12 h postadministration with FI-A(2NI)VE (I), FI-A(2NH2I)VE (II), or FI-A(2NI)VE + dicoumarin (III) (right). Copyright 2021, American Chemical Society.

The construction of supramolecular aggregates *in vivo* for imaging is becoming a frontier research hotspot and has received extensive attention. The controllable living body assembly and disassembly greatly reduces the toxicity of the material in the organism. This novel strategy provides a unique perspective for the design of imaging agents. (Li et al., 2016). In the following text, the latest developments in peptide-based imaging probes are introduced, which included optical imaging, photoacoustic tomography and magnetic resonance imaging (MRI). Recently, Yu and co-workers designed and synthesized an unconventional amino acid that reacts with NTR (nitro-reductase) to regulate the self-assembly of peptides into a supramolecular probe for morphological transformation, which could be used to efficient hypoxia imaging. (Hu et al., 2021). The nitroimidazole part was integrated into the side chain of alanine, the NTR response amino acid was designed, and the nitro position of the nitroimidazole was optimized to obtain 2-nitroimidazole-1-acylalanine with the maximum reduction ability, called A(2NI) (Figure 11A). In addition, the aromatic peptide at the end of Fmoc was used to conduct NTR-responsive SA between nanofibers and nanoparticles (Figure 11B). Fluorescence results and morphological studies were combined into a fluorescent dye functionalized tripeptide IR780-A(2NI)VE to realize tumor cell imaging. Nitroimidazole quenched the fluorescence of the fluorophore through the PET mechanism. When reacting specifically with NTR, the nitro group was reduced to an amino group, causing the fluorescence to be released (Figure 11C). In the end, *in vivo* and *in vitro* fluorescence imaging studies had shown that supramolecular probes had good and efficient fluorescence imaging capabilities in solid tumors in mice (Figure 11D).

**FIGURE 12:**
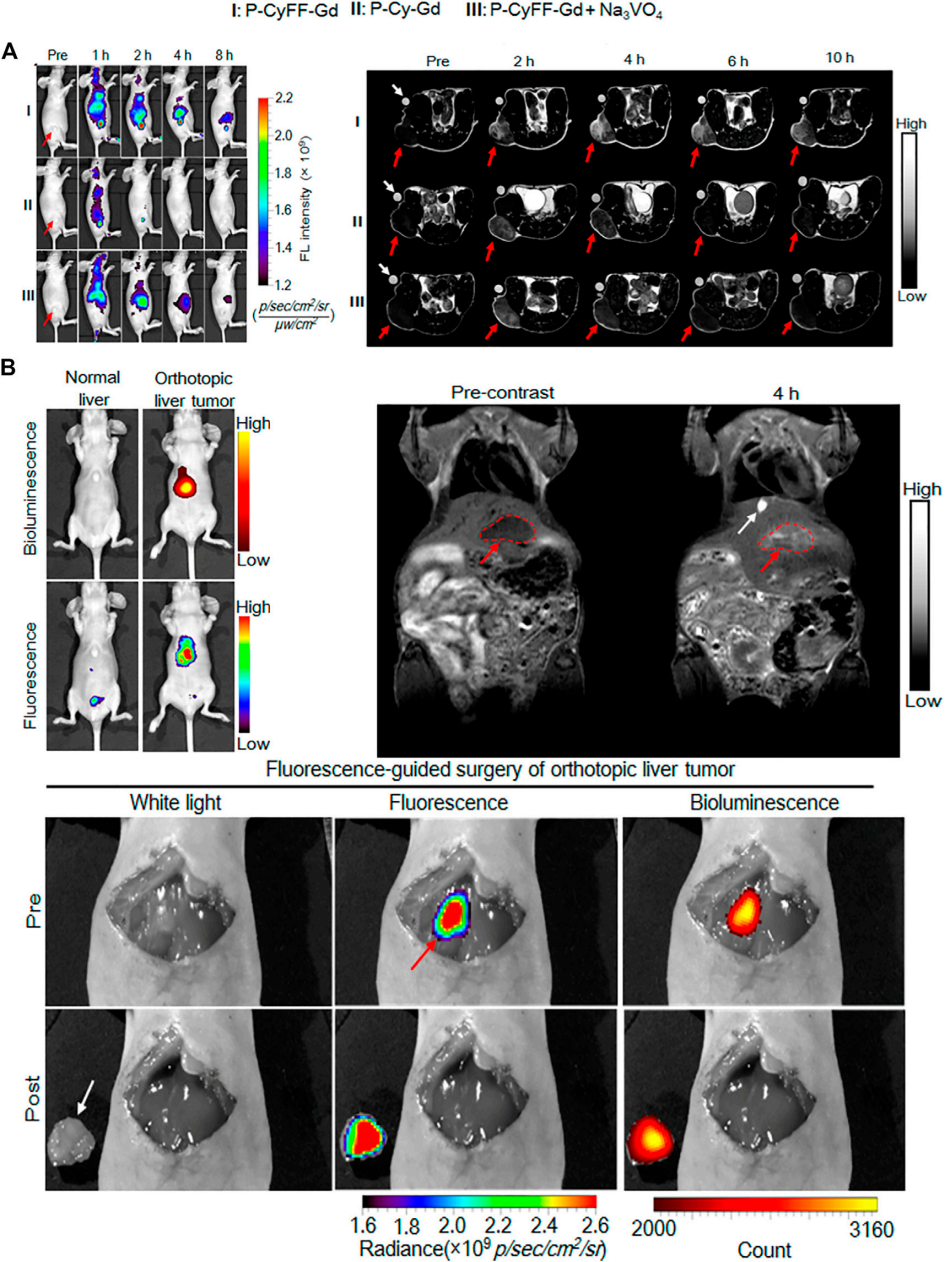
**(A)** Longitudinal FL imaging (left)T1-weighted MR images(right) **(B)** Whole-body fluorescence (down) and bioluminescence (up) imaging of normal mice and orthotopic HepG2/Luc liver tumor xenograft mice. The fluorescence images were acquired in living mice following i.v. injection of P-CyFF-Gd (50 μM, 200 μL) at 4 h (λex/em = 660/710 nm) (top, left) T1-weighted MR imaging of orthotopic HepG2/Luc liver tumor xenograft mice. Images were acquired before (Precontrast) and 4 h post i.p. injection of P-CyFF-Gd (0.015 mmol kg^−1^) at 1 T. Red dotted circles indicate the locations of tumor in liver. White arrow indicates the gallbladder (top, right) Imaging-guided surgical resection of orthotopic HepG2/Luc liver tumor in an intraoperative mouse 30 min after directly spraying P-CyFF-Gd (10 μM) on liver. White arrow indicates the resected tumor tissue, and red arrow indicates the tumor foci in liver tissue detected by NIR fluorescence imaging (bottom). Copyright 2019, American Chemical Society.

In recent years, MRI had become one of the most widely used diagnostic techniques due to its advantages of non-invasiveness, high spatial resolution, and strong tissue penetration. However, compared with other imaging methods, the sensitivity of MRI was relatively low, so multimodal molecular imaging probes had become a trend. (Tang et al., 2020). For example, a dual-modal imaging probe that combined MRI and near-infrared (NIR) fluorescence synergistically, where MRI could produce anatomical images with infinite tissue penetration depth and high spatial resolution, while NIR fluorescence could produce high sensitivity Image. Recently, in one excellent work of Ye and co-workers, they designed and synthesized a small molecule-based NIR fluorescence/MRI dual-modal probe (P-CyFF-Gd) for *in vivo* imaging through *in-situ* self-assembly enzyme-mediated fluorescence reaction. (Yan et al., 2019). The probe (P-CyFF-Gd) could be activated by the endogenous ALP, which was overexpressed on the cell membrane, to produce membrane-localized assembled nanoparticles (NPs). In order to clarify the ability of P-CyFF-Gd to image *in vivo*, the activation effect of ALP on NIR and MR in living mice was studied by subcutaneous injection of P-CyFF-Gd. The resulted show that ALP-mediated fluorescence reaction and *in-situ* self-assembly could effectively activate P-CyFF-Gd and enhance the accumulation of probes in ALP-related tumors, thereby realized high-efficiency fluorescence and MR dual imaging of ALP-related tumors *in vivo*. Considering that ALP-mediated fluorescence response and *in-situ* self-assembly could promote the localization of activated nanoparticles (NPs) in tumor tissues, P-CyFF-Gd was used as a visual guidance for *in-situ* HepG2 liver tumor resection (Figure 12A). Intraoperative mouse bioluminescence imaging confirms successful liver tumor resection (Figure 12B).

**FIGURE 13:**
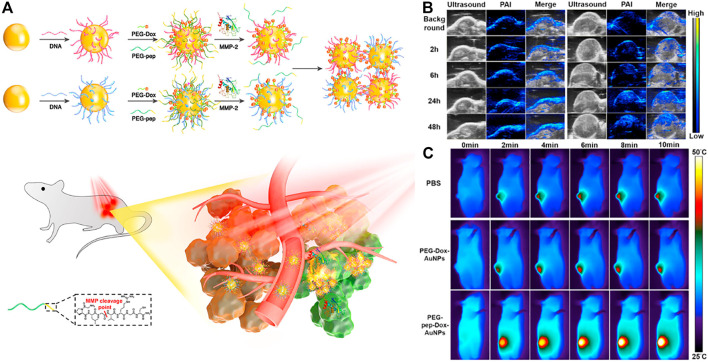
**(A)** Schematic illustration of MMP-induced aggregation of AuNPs *in vivo* for enhanced PAI/PTT of tumor **(B)** Representative PA images of SCC-7 tumors taken before and at different time points after intravenous injection of PEG-Dox-AuNPs (left) and PEG-Pep-Dox-AuNPs (right). The maximum PA intensity was observed at 24 h p.i. in both groups **(C)** Thermographic images of mice under 808 nm irradiation (1 W cm^−2^) at various time intervals 24 h p.i. of PBS, PEG-Dox-AuNPs and PEG-Pep-Dox-AuNPs. Copyright 2019, Biomaterials.

Photoacoustic imaging (PAI or Optoacoustic imaging, OAI) is a non-invasive, low-cost imaging technology that relies on the light absorption of biological tissues that has developed rapidly in recent years. It can make up for the shortcomings of existing imaging modes and provide The obtained diagnostic information, such as tissue oxygenation level, high-resolution vascular network information, etc., which has broad application prospects in the field of biomedicine. (Huang, 2019). Nie group designed a nano-platform, which was based on matrix metalloproteinase (MMP) responsive, for tumor-targeted photoacoustic (PA) imaging guided photothermal therapy. (Yang K et al., 2019). By connecting complementary DNA strands on the surface of the nanoparticles, and then connecting Dox to the heat-labile AuNPs *via* a 4′4-azobis(4-cyanovaleric acid) linker, MMP-responsive AuNPs(PEG-pep-Dox-AuNPs) were designed and prepared (Figure 13A). Using MMP-inert PEG-Dox-AuNPs as a control, the PA performance of MMP-responsive nanoparticles was evaluated. Many small particle areas with strong PA signals could be clearly observed in the tumor tissues treated with PEG-PEP-Dox-AuNPs (Figure 13B). After intravenous injection of the nanoprobe and 808 nm laser irradiation, the local tumor temperature rapidly increased by 17.8°C within 10 min (1 W cm^−2^) (Figure 13C), which was sufficient to inhibit tumor growth *in vivo*, indicating that it enhances the PT efficiency.

## 4 Conclusion and Outlook

Although increasing amount of nanomedicine is being developed and approved in order to reduce side effects and improve efficacy, traditional drugs still dominate drug development pipelines. Nanomedicine should not simply incorporate drugs within nanomaterials, but should rationally design and utilize the nanotechnology based on a deep understanding of biological processes related to diseases. This is why nanomedicine of *in-situ* SA peptides emerged. The activation of *in-situ* SA requires various stimulus from pathological environment and biological processes. Thereby, it exhibits improved precise in targeting and increased intelligence in delivering. However, the clinical translation of this kind of new nanomedicine is still rare. Common challenges of other kind of nanomedicine including safety, scale-up cost and regulation also hindered its development and clinical translation, which needs more efforts from both academic and industrial fields. To promote the clinical translation, researchers should work closely with clinicians to find out the real problems and possible solutions, instead of making more and more complicated formulations. This shift also requires intensive cooperation of material scientists with pharmaceutical companies and regulatory authorities.
